# Rapid Prototyping of Polymer-Based Rolled-Up Microfluidic Devices

**DOI:** 10.3390/mi9100516

**Published:** 2018-10-13

**Authors:** Rerngchai Arayanarakool, Hian Hian See, Samuel David Marshall, Niven Singh Virik, Heng Wang, Poh Seng Lee, Peter Chao Yu Chen

**Affiliations:** Faculty of Engineering, Department of Mechanical Engineering, National University of Singapore, Singapore 119077, Singapore; rerngchaia@gmail.com (R.A.); hhsee@u.nus.edu (H.H.S.); mpesdm@nus.edu.sg (S.D.M.); mpensv@nus.edu.sg (N.S.V.); wangheng@thml.t.u-tokyo.ac.jp (H.W.); pohseng@nus.edu.sg (P.S.L.)

**Keywords:** prototype fabrication, roll-to-roll (R2R) processing, polymeric thin film, microfluidic heat transfer, curved channels

## Abstract

This work presents the simple and rapid fabrication of a polymer-based microfluidic prototype manufactured by rolling up thin films of polymer. The thin films were fabricated via a casting method and rolled up around a center core with the aid of plasma activation to create a three-dimensional (3D) spiral microchannel, hence reducing the time and cost of manufacture. In this work, rolled-up devices with single or dual fluidic networks fabricated from a single or two films were demonstrated for heat sink or heat exchanger applications, respectively. The experimental results show good heat transfer in the rolled-up system at various flow rates for both heat sink and heat exchanger devices, without any leakages. The rolled-up microfluidic system creates multiple curved channels, allowing for the generation of Dean vortices, which in turn lead to an enhancement of heat and mass transfer and prevention of fouling formation. These benefits enable the devices to be employed for many diverse applications, such as heat-transfer devices, micromixers, and sorters. To our knowledge, this work would be the first report on a microfluidic prototype of 3D spiral microchannel made from rolled-up polymeric thin film. This novel fabrication approach may represent the first step towards the development of a pioneering prototype for roll-to-roll processing, permitting the mass production of polymer-based microchannels from single or multiple thin films.

## 1. Introduction

Recently, microfluidic devices have been implemented in a wide variety of applications, ranging from biological analysis to energy harvesting [1,2,3,4]. These devices offer a number of useful capabilities, such as the ability to precisely control fluid, as well as a reduced consumption of samples or reagents. Microchannel-based devices allow for low fabrication and material costs for polymer-based devices and they provide ultra-high surface-to-volume ratio properties, both of which are desirable characteristics for cost-effective improvement of high-efficiency thermal applications [5,6,7,8]. Furthermore, the versatility of microchannel designs permits multi-functionality for the devices. For instance, curved channels can create Dean vortices that are used for size-dependent separation of microparticles and enhancements of mass and heat transfer in the channel [9,10,11,12,13]. In addition, micro-structures (e.g., micro-pillars or herringbone micro-structures) that are integrated into the microchannel can also dramatically improve heat and mass transfer by disrupting the boundary layers of the flowing fluid. 

Despite the aforementioned benefits, the present lack of an adequate method for the mass production and rapid assembly of microfluidic systems for high fluidic flow rates hinders their usage in the industry level. One potential approach for mass production is to fabricate multiple layers of microchannel and later assemble all layers to obtain the final device, however this approach is still time-consuming in all stages of the process i.e., fabrication, alignment, and assembly [14]. This has led to the development of a manufacturing method known as roll-to-roll (R2R) technology for mass producing the thin-film microfluidic devices. This method works by first imprinting the microchannels into a polymeric film and then laminating another layer of polymeric film onto the first to form the microfluidic device [15,16]. However, this method is still expensive, impractical, and rigid (i.e., it makes the fabrication of different designs cumbersome) for small-scale production, especially for prototyping purposes or at the laboratory level. In general, a prototype should be simply and quickly fabricated at a low cost to validate a numerical study or to evaluate the performance of a newly-designed device prior to mass production. The current prototyping approaches reported in the literature for thin-film devices, such as micromachining, xurography, and three-dimensional (3D) printing, can create 3D (out-of-plane) microfluidic networks for facilitating high fluidic flow rates in the device, but still suffer from their low throughput and time-consuming processes [17,18,19]. This has motivated the development of a novel technique for the rapid fabrication of microfluidic device prototypes.

In this paper, we propose a novel and simple fabrication technique to rapidly manufacture a thin-film microfluidic device with 3D (out-of-plane) spiral micro-channels. This technique works by casting a polymeric thin film with patterned microchannels, rolling this thin film around a cylindrical core to close the individual channels and form the 3D spiral structure. Hereafter, the device is referred to as the rolled-up microfluidic device. The chosen material for the film is polydimethylsiloxane (PDMS), so that a simple, fast, and low-cost casting method can be used to pattern the channels, and the device can be assembled by using plasma bonding to avoid the use of adhesive and strong chemicals [20]. Moreover, PDMS provides advantages including chemical resistance and good thermal stability (up to 450 °C), which allows the devices to work in harsh conditions [21]. In this work, the rolled-up polymeric devices were developed by focusing on heat-transfer applications i.e., heat sink and heat exchangers. The rolled-up structures provide several potential benefits, such as the highly-compact design of the device allowing for the usage in a confined space, and increased surface area for high efficiency of heat transfer. Moreover, the rolled-up device contains multiple curved channels, which can generate Dean vortices, which in turn aid in enhancing heat and mass transfer within the channel [11]. Despite the low thermal conductivity of polymer, the thin polymer film would still be beneficial in decreasing thermal resistance, allowing for higher thermal performance [22,23,24].

With the proposed fabrication technique, two different designs of rolled-up devices were made, namely, (i) the single 3D spiral channel device, and (ii) the dual 3D spiral channels device. The single and dual channel rolled-up devices were fabricated from a single thin film or two thin films in order to be employed as a liquid-based heat sink and a liquid-to-liquid heat exchanger, respectively, and for their effectiveness of heat transfer to be characterized. 

To date, only one prior rolled-up microfluidic device by which the microchannels were made by rolling PDMS-parylene thin films has been reported in the literature [25]. However, the method used was a chemical vapor deposition technique (CVD), which would not be suitable for large scale microfluidic systems or mass production of the devices, as the size of the CVD machine will limit the scale of the device. Said microchannels was also employed solely as a particle-separator, not as a heat sink or exchanger. As such, this current work would be the first report on a scalable microfluidic prototype of 3D spiral microchannels fabricated by rolling up polymeric thin film(s). This novel fabrication approach could represent an initial step in making a pioneering prototype for a polymer-based roll-to-roll processing, allowing for the mass production of the polymer-based microchannels in form of thin films. Our work was developed by focusing on the usage of polymer-based thin film for further development of R2R technology, especially for cases where rapid and simple prototyping of the device is required to validate transport phenomena, such as heat and mass transfer, in the new design of a microfluidic network. For instance, in our previous work, simulations were performed to optimize the dimensions of a microchannel rolled-up heat exchanger that is made from thin film polymers, demonstrating that the effects of curved microchannels can highly improve heat transfer performance when compared to an equivalent straight channel [25]. This current work would then be a promising approach for fabricating a prototyping device in order to validate that kind of numerical study. In addition, regarding the device itself, Dean vortices are generated along the curved channel, which can be exploited for the size-dependent separation of microparticles as well as the prevention of fouling in a microchannel [12,26]. These rolled-up devices thus can be used for diverse applications, such as heat sinks, heat exchangers, micromixers, micro separators, and desalination equipment [22,23,24,25,26,27,28]. 

## 2. Materials and Methods

### 2.1. Fabrication of the Rolling-Up Device

**Figure 1:**
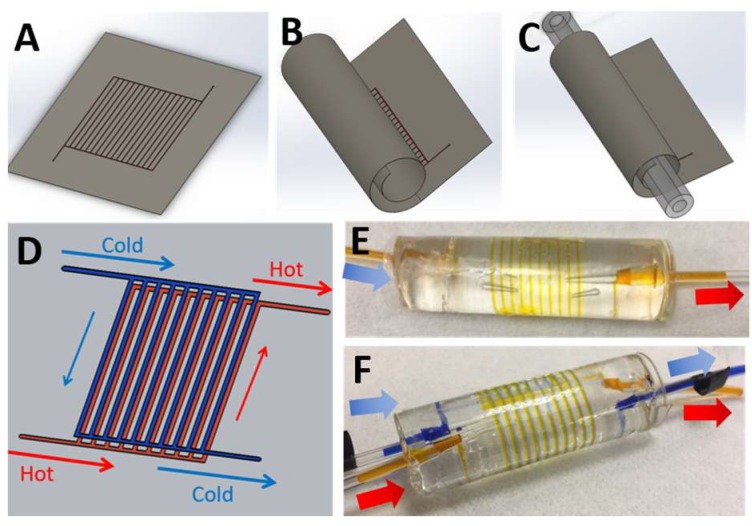
The illustration of the roll-up microfluidic system made from polymer thin film. (**A**) The thin-film microfluidic system was made by casting polymeric resin (PDMS) onto the mold containing the microfluidic channels. (**B**) After curing, the PDMS thin film was flexible enough to be rolled up around the PDMS center core, also made via casting. (**C**) The thin-film and center core were bonded together by using oxygen plasma activation to obtain the three-dimensional (3D) spiral microchannel. This method allows for the creation of multiple fluidic layers by rolling multiple microchannel thin films (i.e., hot-water layers and cold-water layers) around the core, thereby forming a heat exchanger device with two fluidic streams. The dual-channel device contains hot and cold fluid running in a counter-cross flow arrangement, as shown by the red and blue arrows for hot and cold water streams, respectively. (**D**) The rolled-up microfluidic device with single layer of microchannel was fabricated and demonstrated as a heat-sink application. (**E**) The rolled-up microfluidic device with two microchannel layers was employed as heat exchanger device. (**F**) The arrows indicate the fluidic direction inward or outward relative to the device. The diameter of the center core was approximately 20 mm.

The microchannels were designed by using the modelling software SolidWorks to make molds for casting the polymer replica. The metallic molds were fabricated by using a milling method (Whits Technology, Singapore). The polymeric thin films with microchannels were made from PDMS, which was fabricated via soft lithography [29]. Firstly, the PDMS resin was made by mixing a precursor and curing agent with the mass ratio of 10 to 1. The mixed solution was then poured onto the mold and degassed to remove all the air bubbles in the solution, prior to curing at 120 °C for one hour. Afterward, the polymer thin film was gently peeled off from the mold. The center core was fabricated via the same method. Next, the two components were activated by using oxygen plasma (Femto Science, Gyeonggi-Do, South Korea) prior to aligning and rolling a thin film around the core to obtain the final device, as illustrated in Figure 1A–C. Finally, PFA tubings (Monotaro, Amagasaki, Japan) with an outer diameter of 3 mm were attached to the two ends of the center core, in order to connect the device to the experimental setup and keep pressure drop at a minimum. PFA tubings are chemical resistant, translucent, and can work under both high pressure (up to 18 bar) and high temperature (up to 200 °C). 

### 2.2. Rolled-Up Microfluidic Device

The microfluidic networks with parallel channels were constructed so as to provide increased heat transfer area whilst still maintaining a reduced pressure drop and avoiding the risk of channel collapse that is caused by a high aspect ratio microchannel (see Figure 1A). The width of the channel was 1 mm, whilst the heights of the channels were approximately 500 µm or 100 µm for the device constructed from a single thin film or two thin films, respectively. Each device consists of ten parallel channels with a branch length of 55 mm. To ensure the uniformity of the thin film, and to avoid stretching and wobbling of the film as it is peeled off the mold and bonded to the core (especially in the smaller dual-channel device), the thickness of the film should be at least twice the height of the channel. Hence, in the current case, the films employed were 1000 µm or 200 µm for the single thin film or dual-channel thin films, respectively. It should be noted that, though the high elasticity of PDMS offers the possibility to roll up the PDMS film, thick PDMS film can create difficulties during rolling up around the small core. In the current case, the PDMS film with a thickness greater than 1 mm cannot be rolled up around a center core with diameter of 20 mm without suffering from fractures or cavities. Due to this limitation, the dual-channel device that was constructed from two layers of microchannel thin films was required to be have reduced thickness compared to the single channel device (down to 100 µm from 500 µm). After removing the PDMS replicas from the molds, the thin film and the center core were activated by using oxygen plasma. With the plasma activation, PDMS samples can be bonded together at room temperature without an intermediate layer, such as adhesive, which can lead to clogging in the microchannel. This plasma-aided bonding of PDMS allows for bonding of delicate structures, such as small microchannels. The final state of the devices with fully rolled-up microfluidic channels around the center core made from a single thin film and two thin films are shown in Figure 1E,F, respectively. 

In addition, to achieve a good alignment of the two microfluidic thin films, it was found to be beneficial to peel off only one film, whilst the other was still attached onto the master mold. After bonding both thin films together, the bonded films were then peeled off from the mold and attached to the center core. Moreover, to avoid the issue of the “step” formed by the edge of the film when rolling up, the film was designed with empty space without microchannel elements at the edge. This extra space was firstly rolled around the center core for at least one revolution to create a uniform layer of the film around the core, before rolling up the rest of the film with the microchannel around it. Since the interface between access holes on the film and the fluidic pathway in the center core is critical, it is recommended that, upon pre-bonding, the film is first punched to create the access hole, and then carefully aligned with the center hole. A needle tip can then be used to create a small connection between the access hole in the film and the pathway in the center core. The puncher can subsequently utilize again to produce larger connecting holes. After cleaning all debris, the thin films and the center core were activated by plasma. The film was vertically tiled in order to uniformly activate both side of the film upon plasma treatment. By this methodology, the successful rate of fabrication for both device types was around 80%. It is believed that this value can be greatly improved with superior materials and further refinement of the manufacturing process.

The microfluidic network in the thin film contains multiple parallel microchannnels that are split from one main channel. After rolling up, the rolled-up microfluidic system contains 3D multiple curved microchannels that are connected together via the shared inlet. For the dual-channel design, the device was made by rolling two layers of the microchannel thin film around the center core to create two fluidic networks flowing around the core–the first layer for the hot fluidic stream and the second for the cold fluidic stream, as illustrated in Figure 1D,F. To promote effectively heat transfer, the dual-channel device contains hot and cold fluid running in a counter-cross flow arrangement, as shown by the red and blue arrows for hot and cold water streams, respectively.

In addition, due to the transparency of the PDMS material, the quality of bonding can be simply visualized upon the completion of fabrication, as well as any possible leakage. The casting method allows for simple, fast, and cost-effective manufacture of devices with varying dimensions. In this current work, the fabrication approach was demonstrated with a 12 cm by 14 cm PDMS thin film and a PDMS core with diameter of 20 mm, resulting in a final device with dimensions of 14 cm by 2 cm, as shown in Figure 1E,F. Moreover, this rolled-up approach allows for the creation of 3D multiple spiral microchannels along the center core with reduced time and cost of fabrication, as compared with a conventional approach, such as multiple flat microchannels assembled together to create 3D microfluidic system.

### 2.3. Thermal Performance Test

**Figure 2:**
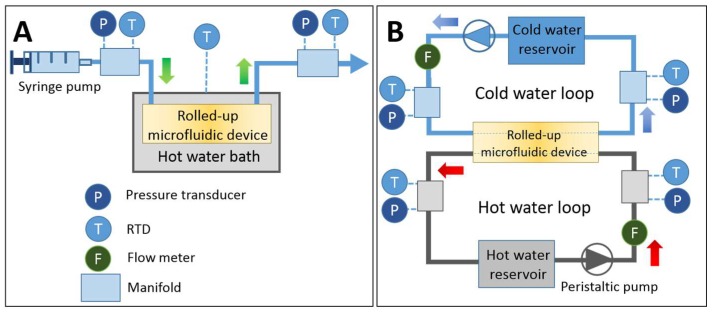
Illustration of the experimental setup. (**A**) The setup for the single-channel device consisted of one syringe pump to inject DI water into the device whilst the device was immersed in the warm water bath. While running through the rolled-up device, the running DI water harvested the heat flux from the hot water bath at varied flow rates, resulting in increasing temperature at the outlet. (**B**) The setup for dual channel device contains two loops of fluids i.e., hot water stream and cold water stream. Each loop had a peristaltic pump and water bath to set the flow rates and temperatures. The inlet and outlet temperatures were measured by using resistance temperature detector (RTD) probes and pressures were measured by pressure transducers, both of which were connected to a data acquisition system (DAQ).

In this study, preliminary fluidic tests were conducted in order to validate the leakage-free system and determine the heat transfer characteristics of the fabricated device. The rolled-up microfluidic devices were employed for the application of thermal transport i.e., the single-channel device was used as a heat sink and the dual-channel device was used as a heat exchanger. In the single-channel experiment, the device was submerged into a Heated Circulating Bath (PolyScience, Niles, IL, USA) with a controlled temperature of 50 °C, which was used as a heat source. The experiment was conducted by injecting a circulating fluid (DI water) into the device at a constant flow rate by using a Legato 200 Dual Injection Syringe Pump (KD Scientific, Holliston, MA, USA) to harvest the heat from the water bath. The temperature and pressure of the fluid were measured both upstream and downstream of the device, using OMEGA Ultra Precise Resistance Temperature Detector (RTD) Sensors and OMEGA pressure transducers connected to a LabVIEW control and data acquisition system (DAQ). The accuracy of the RTD sensors was approximately 0.1 °C. The schematic of the experimental setup is shown in Figure 2A.

In the case of the dual-channel test, the device was mounted into a setup consisting of both a hot water loop and a cold water loop (see Figure 2B). Hot and cold water streams were separately injected into the system, so that the heat can transfer from the hot channel to the cold channel via the rolled-up device. Each loop consisted of a Masterflex L/S Peristaltic Pump (Cole-Parmer, Vernon Hills, IL, USA) to create a uniform flow of DI water, a Remote Liquid Flow Meter (Alicat Scientific, Tucson, AZ, USA), the RTD sensors (OMEGA, Norwalk, CT, USA), pressure transducer (OMEGA, Norwalk, CT, USA), and a Refrigeration Bath Circulator (Thermo Fisher Scientific, Waltham, MA USA) with temperature control used as a water reservoir. To simplify the effects of fluidic flow in both the hot water and cold water loops in this preliminary test, the flow rate of the cold loop was kept constant at 1.3 mL/min. In this way, the independent effect of altering the flow rate of the hot loop on the heat-transfer characteristic of the device could be observed.

In the single-channel heat sink experiment, the inlet temperature of the DI water was chosen to be 300 K, so that the DI water would be injected into the device at close to room temperature. For the heat exchanger experiment, the inlet temperature of the cold loop was also selected as 300 K, for same reason. On the other hand, the hot loop inlet temperature ranged almost linearly from 305 K for the lowest flow rates up to 330 K for the highest flow rates. This range was caused by the heat loss along tubing between the heat source (the hot water bath) and the device. At the slower flow rates, a longer residential time of hot water in contact with the tubing resulted in lower temperatures at the inlet of the device.

### 2.4. Characterization of Thermal Performance

The temperatures and pressures of the fluid measured upstream and downstream of the devices were used to determine the thermal performance of the devices. The heat transfer characteristics of the device were expressed in terms of temperature change, pressure drop, Nusselt number, and thermal performance factor (TPF). The Nusselt number, the ratio of convective heat to conductive heat transport in a fluid, can be expressed as:

(1) $$Nu=\frac{D_{h}\dot{m}C_{p}\left(T_{f,in}-T_{f,\;out}\right)}{kA}\times \frac{ln\left(\frac{T_{w}-T_{f,in}}{T_{w}-T_{f,\;out}}\right)}{\left(T_{w}-T_{f,in}\right)-\left(T_{w}-T_{f,\;out}\right)}$$

where *D_h_* is the hydraulic diameter of the microchannel, *ṁ* is a mass flow rate of the fluid, *C_p_* is the specific heat capacity of the fluid, *T_f, in_* is the temperature of inlet fluid, *T_f, out_* is the temperature of outlet fluid, *T_w_* is the temperature of the channel wall, *k* is the thermal conductivity of the fluid, and *A* is the heat transfer area. Similarly, *f* is the friction factor derived from Darcy–Weisbach equation as:

(2) $$f=\frac{2D_{h}\Delta P}{L\rho U^{2}}$$

where ∆*P* is the pressure drop across the microchannel, *L* is the length of the channel, *ρ* is the density of the fluid, and *U* is the fluid flow velocity. On the basis of a constant consumed energy of pumping power, the thermal performance factor (TPF) can be derived from the Nusselt number and the friction factor as:

(3) TPF=Nuf13

The thermal characteristics of the devices were plotted against the Reynolds number, as determined from the equation below:

(4) $$Re=\frac{\rho D_{h}U}{v}$$

where *ν* is the viscosity of the fluid. 

## 3. Results and Discussion

### 3.1. Preliminary Test of Thermal Performance of Rolled-Up Device as a Heat Sink

**Figure 3:**
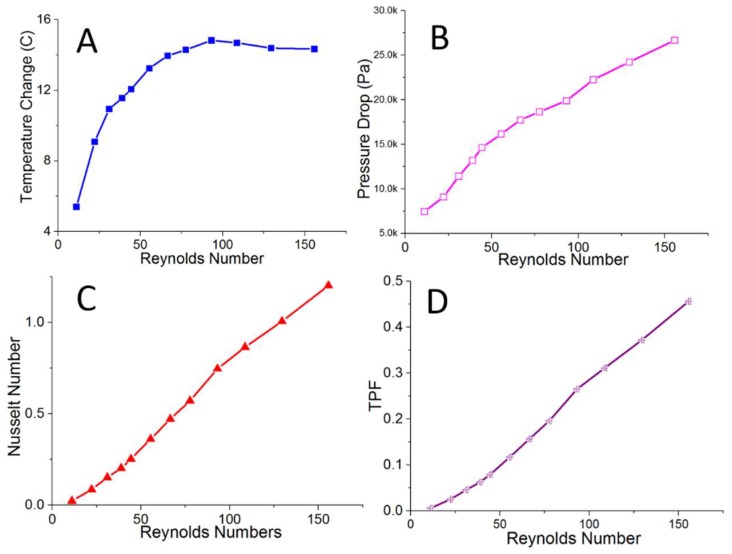
The experimental results of the single-channel rolled-up microfluidic system. The device was immersed into a warm water bath at constant temperature of 50 °C, as illustrated in Figure 2A. The flow rates of DI water in the microchannel were varied to determine the thermal performance of this device. (**A**) The temperature change of water was plotted against the Reynold number. (**B**) The pressure drop along the channel was measured at varied flow rates. The thermal performances of the rolled-up device, determined via Nusselt number (Nu) and thermal performance factor (TPF) (**C**,**D**), respectively. In this thermal test, the fluidic flow rates were varied from 0.5 to 7 mL/min, corresponding to Re of 10 to 160.

The results of the testing of the rolled-up device employed as a heat sink are shown in Figure 3. The single-channel experimental results demonstrate that when DI water flows through parallel curved channels along the rolled-up device, the heat from the hot water reservoir is harvested, resulting in a temperature change between the inlet and outlet of up to 15 °C (see Figure 3A). This temperature change increases with rising flow rate–steeply at Re < 50 and more slowly after that—until it reaches a maximum (around 15 °C) at approximately Re = 125, after which it stabilized at that value. As would be expected, the pressure drop increases proportionally with flow rate, as shown in Figure 3B. The lack of sudden drops or peaks in this measurement, combined with the absence of bubbles of other visual signs of water loss, verify that the rolled-up device can be assumed to free of leakage. Much like for the pressure drop, the Nusselt number (Nu) also increases with the flow rate, with no observable maximum value within the parameter space explored, unlike for the temperature change (see Figure 3C). As both the pressure drop and the Nusselt number form roughly linear relationships with Reynolds number, so too does the thermal performance factor (TPF), as demonstrated in Figure 3D. This being the case, these experimental results verify that, despite the low thermal conductivity of the material employed, our rolled-up device can provide a prototype that features good thermal performance, which is suitable for heat-recovery applications. 

The rolled-up devices were also tested to verify the limits of their leakage-free behaviour. In this study, it was found that the single-layer device can withstand high pressures of up to 80,000 Pa before internal leakage between microchannels, which is revealed via a sudden drop or rise of the pressure inside each channel, as monitored in real-time by LabVIEW. In the future, tougher materials would be able to significantly improve this limit. 

In a review of the available literature, only a few reports were found demonstrating the thermal performance of a microchannel-based device constructed from polymer. As would be expected, when compared with a metal-based heat sink [30], the thermal performance of our polymer-based thin film device is significantly lower, due to the fact that the thermal conductivity of copper is 3000 times higher than that of PDMS. These metal-based devices also operate at notably higher flow rates. However, when compared with another device made from the same material as our device and with comparable microchannel dimensions [31], the thermal performance of our heat sink (maximum Nu of approximately 1.2) is comparable to the heat sink described in that work (maximum Nu of approximately 1.7).

### 3.2. Preliminary Test of Thermal Performance of Dual-Channel Rolled-Up Device as a Heat Exchanger

**Figure 4:**
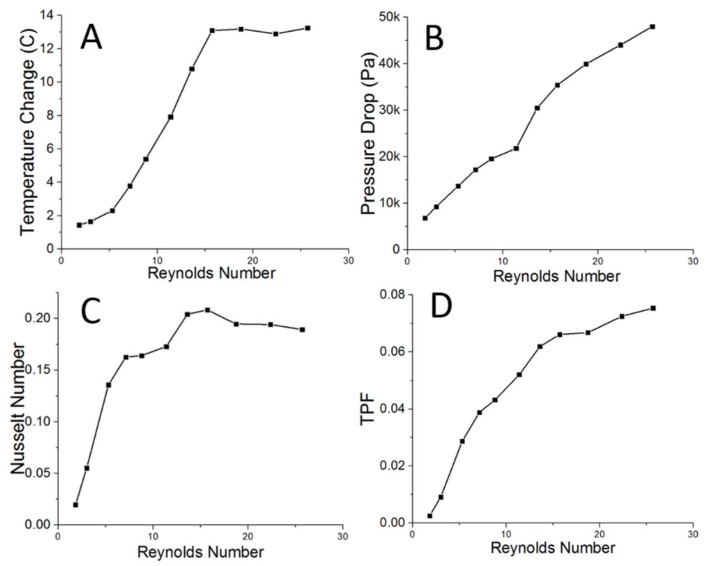
The experimental results of the dual-channel rolled-up microfluidic system. The device was mounted into the two-loop experimental setup shown in Figure 2B. The hot water and cold water were separately injected into a hot channel loop and cold channel loop in the rolled-up device, respectively. The change in temperature of the hot water stream was plotted against the Reynolds number (**A**) showing good heat transfer between hot and cold channels. (**B**) The pressure drop was plotted against the Reynolds number, showing no leakage in our device. (**C**) The thermal performances of the dual-channel rolled-up device were determined via the Nusselt number and thermal performance factor (TPF), which are plotted against Reynolds number as shown in (**C**,**D**), respectively. In this thermal test, the fluidic flow rates were varied from 0.6 to 8.5 mL/min corresponding to Re of 2 to 26.

The results of the testing of the rolled-up device employed as a heat exchanger are shown in Figure 4. For the preliminary test of the dual-channel device, the flow rate in the cold water loop was keep constant in order to observe the effect of the flow rate of the hot loop on the thermal performance of the device. As can be seen in Figure 4A, at a given constant cold loop flow rate, the water in the hot loop can be cooled down by up to 8 °C between inlet and outlet. Unlike for the heat sink experiments, there was no maximum temperature change or levelling-off observed in this test, though this could possibly be due to the lower flow rates that were employed (required due to the increased pressure from the peristaltic pumps and smaller channel size). The relatively linear plot of pressure drop against the Reynolds number in Figure 4B again shows no evidence of water leakage within the device, verifying that the dual-channel rolled-up device is also leakage-free. This preliminary result shows that heat transfer can efficiently occur between hot and cold channels, despite the low thermal conductivity of the material employed, thus suggesting that the dual-channel rolled-up device prototype made via our approach can also be used effectively for heat-transfer applications. 

Like for the single-layer device, the two-layer device was also tested for the maximum conditions that it could remain leakage-free. For this device, the thin film was able to withstand up to 90,000 Pa before suffering internal leakage between hot and cold channels. As for the heat sink, stronger polymers would be able to increase this maximum survivable pressure.

In order to assess the thermal performance of our two-layer device employed as a heat-exchanger when compared to the literature, another device that is made from the same material and with comparable microchannel dimensions must be utilised. In this way, the heat transfer efficiency of the dual-channel thin film (maximum Nu of approximately 0.2) is found to again be similar to the performance of the two-layer heat exchanger from the same study used previously to compare the heat sink (maximum Nu of approximately 0.3) [31].

### 3.3. Fabrication Techniques and Implications

As discussed, our fabrication approach of microfluidic system by rolling up polymeric thin films can provide several advantages in many aspects. First, our proposed fabrication technique allows for a simple and fast manufacturing process, starting from casting of the thin film, to alignment, and finally assembly of the device. In the conventional approach, the multilayers of microfluidic samples are separately made prior to layer-by-layer assembly of the device. In our approach, the device can be made from a single step of rolling up the large microfluidic thin film. This is significantly less time-consuming than the conventional assembly method. Moreover, the manufacturing cost of polymeric thin films in term of a labor cost (manufacturing time) and the cost of materials is lower than conventional materials (i.e., metal), allowing for further development of the cost-effective mass production of the microfluidic system in the future. As compared with metallic materials, some properties of polymeric materials, such as biological and chemical resistance, light-weight, and transparency, have beneficial qualities. For instance, polymers can be used for the fabrication of devices that are used in harsh conditions, such as environments in contact with high acidic or basis solutions, or seawaters. Additionally, in the aerospace or offshore industries, equipment with highly-compact design, low-footprint area and low weight are strongly advantageous. Thin-film polymer-based devices would be promising for these applications due to the material properties of polymers. Furthermore, the transparency of the polymer can allow for optical observation of the fluid inside the device, for instance, permitting straightforward observation of clogging or fouling inside the device.

In terms of thermal transport performance, when the thin film is rolled up around the center core and the multiple curved channels are created, generation of secondary flow in the form of Dean vortices will occur along the microchannel. In addition, the microchannel design can dramatically increase the surface area per volume ratio of the fluid, and thus the thin film of the polymer can provide decreased thermal resistance along the film, despite the low thermal conductivity of the polymer. These characteristics are all greatly beneficial for heat transfer. In this way, the thin-film microfluidic system is well suited to be employed for heat transfer applications, such as heat sinks and heat exchangers. For example, Heng et al., [26] proposed a polymer-based heat exchanger made from polymeric thin films. They optimized the parameters of the device (i.e., the thickness of the film, dimensions and configuration of the microchannels) to achieve highly-efficient heat transfer in the microchannel by using computational fluid dynamic simulations in the software FLUENT. Our proposed fabrication approach could be applied to produce the rapid prototype of that system to validate experimentally that numerical work. 

Lastly, via our fabrication approach, the rolled-up device can be manufactured from multilayers of polymeric thin film. The multilayered rolled-up microfluidic device containing multiple parallel curved microchannels allows for high fluidic flow through the device while maintaining reduced pressure drop along the system. This principle would be promising for further development of the rolled-up microfluidic system for industrial level usage. As mentioned previously, the rolled-up microfluidic system consists of multiple curved channels that generate Dean vortices, boosting the mass and heat transfer in the microchannel. This principle vortices-induced enhancement of mass and heat transfer in the curved channel has been well-explained and applied for numerous applications, such as mixers, microsorters, waste heat recovery, and heat exchange. In this work, the devices made by using this rolling-up technique were employed as a heat sink to achieve heat recovery and a heat exchanger to achieve thermal transport. For future work, the rolled-up device could be developed and employed for other applications, including micromixers and cell/microparticle separation.

## 4. Conclusions 

This work presents the fabrication of a new prototype system made of rolled-up polymeric thin film, with validation of its heat-transfer properties. The production of the microfluidic device is simple and feasible and the principle of this fabrication approach can be further developed for making multi-layer microfluidic thin films that can facilitate high fluidic flow. Due to the creation of the multiple curved channels from this fabrication approach, the rolled-up devices can be employed for diverse applications, such as heat sinks, heat exchangers, micromixers, microparticle separators, etc. In all cases, the Dean vortices that the curved channels generate act to enhance heat and mass transport relative to an equivalent straight channel. The novel fabrication approach reported in this work may represent the first step towards the development of a pioneering prototype for polymer-based roll-to-roll (R2R) processing, permitting the mass production of polymer-based microchannels from single or multiple thin films.
